# Supplementary material for: Backtracking during navigation is correlated with enhanced anterior cingulate activity and suppression of alpha oscillations and the ‘default-mode’ network
Source: Proc Biol Sci. 2019 Jul 31;286(1908):20191016. doi: 10.1098/rspb.2019.1016 (PMC6710605; doi:10.1098/rspb.2019.1016)
Supplement: fMRI activations table [file rspb20191016supp1.pdf]

Table 2: Summary of all fMRI activations

FWE corrected, min. 5 contiguous voxels

Table shows all local maxima separated by more than 20 mm. Regions were automatically labeled using the AnatomyToolbox atlas using BSPMVIEW https://github.com/spunt/bspmview [https://zenodo.org/badge/latestdoi/21612/spunt/bspmview]

Contrast Name	Region Label	Extent	t-value	z-value	x	y	z
Backtracking > Non-backtracking	R Linual Gyrus	179	10.815	6	9	-79	-4.00
	R MCC	26	9.938	6	6	20	35.00
	L Linual Gyrus	18	9.025	6	-15	-61	8.00
	L Posterior-Medial Frontal	15	9.003	6	-18	-4	71.00
	L Postcentral Gyrus	26	8.322	5	-42	-31	56.00
	R Posterior-Medial Frontal	6	8.017	5	15	-1	71.00
	Location not in atlas	8	7.950	5	24	-58	20.00
	L Cerebelum (VI)	5	7.709	5	-9	-79	-10.00

p<0.001 (uncorrected), min. 5 contiguous voxels

Contrast Name	Region Label	Extent	t-value	z-value	x	y	z
Backtrack > Non-Backtrack	R Linual Gyrus	3210	10.815	6.14	9	-79	-4
	R MCC	2328	9.938	5.91	6	20	35
	Location not in atlas	46	7.065	4.95	-15	-28	38
	R Inferior Parietal Lobule	591	6.958	4.90	42	-40	53
	R Precuneus	102	6.522	4.72	6	-49	53
	R Middle Frontal Gyrus	153	6.213	4.58	33	38	35
	Location not in atlas	73	6.180	4.57	18	-22	-4
	R IFG (p. Orbitalis)	196	5.938	4.46	33	20	-7
	L Middle Frontal Gyrus	106	5.713	4.35	-30	32	29
	Location not in atlas	102	5.637	4.31	-9	-16	2
	R Cerebelum (VIII)	59	5.564	4.28	27	-58	-55
	Location not in atlas	6	4.866	3.91	-9	-34	-34
	L IFG (p. Orbitalis)	10	4.358	3.61	-27	20	-13
	L Cerebelum (Crus 1)	9	4.147	3.48	-45	-58	-34
	L Insula Lobe	12	3.987	3.38	-36	11	-4
Backtrack < Non-Backtrack	Location not in atlas	69	6.163	4.56	-21	-49	32
	L Hippocampus	19	5.448	4.22	-36	-34	-1
	R Insula Lobe	17	5.119	4.04	36	-16	23
	L Angular Gyrus	65	5.034	4.00	-48	-67	32
	R Precuneus	10	4.767	3.85	6	-52	26
	L PCC	9	4.319	3.59	-6	-40	35
	L Mid Orbital Gyrus	18	4.217	3.52	-6	62	-4

	Location not in atlas	5	4.168	3.49	-30	-49	26
	R Mid Orbital Gyrus	5	3.899	3.32	6	32	-7
Backtrack > Turn	R MCC	120	9.069	4.91	9	20	35
	R Linual Gyrus	2313	8.384	4.81	15	-61	8
	Location not in atlas	614	8.147	4.80	24	-1	50
	R SupraMarginal Gyrus	471	7.648	4.69	63	-28	44
	L Cerebelum (IX)	86	7.296	4.65	-12	-58	-52
	L Middle Frontal Gyrus	301	7.245	4.60	-24	-10	53
	Cerebellar Vermis (9)	63	7.068	4.55	3	-58	-34
	L Inferior Parietal Lobule	472	6.891	4.42	-39	-37	44
	R Cerebelum (IX)	88	6.815	4.23	15	-55	-49
	Location not in atlas	23	6.676	4.20	12	-16	-4
	R Cerebelum (VI)	72	5.931	4.11	33	-52	-31
	Location not in atlas	21	5.544	3.99	-12	-22	38
	R Middle Frontal Gyrus	85	5.501	3.89	36	35	26
	Location not in atlas	33	5.480	3.81	-12	-16	-7
	R Thalamus	14	5.382	3.80	9	-16	14
	L IFG (p. Opercularis)	19	4.886	3.77	-45	8	17
	L Insula Lobe	11	4.815	3.76	-39	14	-4
	L Cerebelum (VIII)	6	4.812	3.71	-24	-58	-52
	L Thalamus	20	4.720	3.70	-12	-13	8
	L Insula Lobe	5	4.411	3.66	-27	23	5
	L Precentral Gyrus	5	4.335	3.53	-51	2	35
	R MCC	8	4.234	3.50	12	-28	38
	L Middle Frontal Gyrus	21	4.176	3.49	-39	38	26
	L Cerebelum (Crus 1)	25	4.032	3.45	-30	-67	-31
Backtrack <Turn	Location not in atlas	82	6.410	4.67	15	-28	59
	L Middle Temporal Gyrus	97	6.283	4.62	-42	-58	23
	R Rectal Gyrus	279	6.055	4.51	6	26	-16
	R Olfactory cortex	27	6.010	4.49	9	8	-10
	L Middle Temporal Gyrus	70	5.916	4.45	-60	-7	-10
	L PCC	144	5.904	4.44	-6	-52	23
	L Superior Medial Gyrus	31	5.901	4.44	-3	50	44
	Location not in atlas	34	5.857	4.42	33	-16	23
	Location not in atlas	23	5.752	4.37	21	35	-1
	R Caudate Nucleus	35	5.404	4.19	21	-7	29
	L Superior Frontal Gyrus	16	5.370	4.18	-12	65	11
	L Hippocampus	79	5.328	4.16	-36	-31	-4
	Location not in atlas	8	5.257	4.12	0	-7	8
	Location not in atlas	5	5.140	4.06	-21	-49	32
	R Thalamus	10	5.030	4.00	15	-37	8
	L Middle Temporal Gyrus	91	4.936	3.95	-51	-31	5
	Location not in atlas	15	4.918	3.94	12	-16	-19
	L IFG (p. Orbitalis)	21	4.916	3.93	-45	35	-7
	R Medial Temporal Pole	42	4.910	3.93	60	2	-10

	L Superior Frontal Gyrus	32	4.871	3.91	-15	41	53
	R Hippocampus	13	4.806	3.87	39	-22	-13
	R Angular Gyrus	11	4.692	3.81	54	-64	32
	L Rolandic Operculum	5	4.502	3.70	-39	-22	23
	R Superior Medial Gyrus	17	4.448	3.67	9	65	8
	R Temporal Pole	23	4.440	3.66	60	-1	5
	R Cerebelum (Crus 2)	6	4.440	3.66	36	-79	-37
	Location not in atlas	12	4.410	3.64	39	-31	-10
	L Thalamus	10	4.306	3.58	-12	-34	8
	L Middle Temporal Gyrus	5	4.252	3.55	-54	-19	-1
	L Medial Temporal Pole	10	4.168	3.49	-42	14	-34
	L Middle Temporal Gyrus	5	3.904	3.33	-54	-1	-22
	R Superior Temporal Gyrus	5	3.893	3.32	48	-7	-4
Backtrack > Turn (from backtracking trials)	L Linual Gyrus	1855	10.726	6	-9	-73	-1
	L Postcentral Gyrus	661	6.730	5	-42	-37	47
	Location not in atlas	514	6.658	5	27	-7	47
	R SupraMarginal Gyrus	432	6.453	5	54	-31	47
	Location not in atlas	46	5.998	4	-12	-25	38
	R MCC	55	5.888	4	6	-19	38
	Location not in atlas	23	5.866	4	9	-16	-10
	L Middle Occipital Gyrus	55	5.637	4	-42	-73	11
	Location not in atlas	86	5.569	4	18	-55	-25
	R Cerebelum (IX)	35	5.555	4	15	-61	-46
	L Cerebelum (IX)	17	5.550	4	-15	-49	-52
	Location not in atlas	5	5.188	4	6	17	20
	Location not in atlas	10	5.084	4	-24	-40	-49
	R Cerebelum (VIII)	10	5.049	4	27	-43	-49
	L Precuneus	7	5.018	4	-12	-49	53
	R IFG (p. Opercularis)	70	4.997	4	48	14	11
	Location not in atlas	22	4.877	4	-12	-13	-1
	L Thalamus	24	4.851	4	-12	-19	11
	L Cerebelum (IX)	42	4.851	4	0	-58	-40
	R Middle Frontal Gyrus	36	4.711	4	36	41	29
	L IFG (p. Orbitalis)	15	4.644	4	-27	20	-10
	L Cerebelum (VI)	10	4.635	4	-24	-58	-22
	R Thalamus	7	4.617	4	12	-10	5
	L IFG (p. Opercularis)	9	4.611	4	-42	11	17
	L Cerebelum (VIII)	5	4.437	4	-24	-70	-37
	L Middle Frontal Gyrus	10	4.234	4	-39	38	32
	L Cerebelum (VII)	13	4.187	4	-33	-40	-34
	Location not in atlas	7	4.166	3	30	26	-1
	R Precentral Gyrus	5	4.091	3	48	5	50
	R Superior Frontal Gyrus	5	4.005	3	21	59	23
	R Fusiform Gyrus	5	3.979	3	21	-46	-7
	R Precentral Gyrus	5	3.962	3	57	5	38
	L Calcarine Gyrus	5	3.937	3	-18	-67	20

	Location not in atlas	9	3.789	3	-21	-70	-52
Backtrack <Turn (from backtracking trials)	L PCC	12	5.751	4	-9	-52	26
	Location not in atlas	13	5.716	4	33	-46	20
	R Caudate Nucleus	11	5.504	4	21	-7	29
	Location not in atlas	5	4.435	4	-33	-46	17
	L Mid Orbital Gyrus	5	4.340	4	-6	56	-7
	L Angular Gyrus	6	4.204	4	-42	-61	29
	L Hippocampus	7	4.145	3	-33	-31	-4
Turn > Non-Turn	R Linual Gyrus	6453	15.501	7	6	-82	-4
	L Precentral Gyrus	2961	13.463	7	-42	-16	59
	L Thalamus	290	11.281	6	-12	-19	5
	R Thalamus	46	7.466	5	12	-19	8
	Location not in atlas	49	6.423	5	6	-28	-34
	Location not in atlas	10	5.539	4	9	-16	-10
	R SupraMarginal Gyrus	31	5.388	4	57	-31	23
	R Paracentral Lobule	24	5.097	4	15	-43	53
	Location not in atlas	21	4.962	4	21	-28	-1
	R Rolandic Operculum	19	4.891	4	39	-31	26
	Location not in atlas	5	4.747	4	36	-25	38
	R Postcentral Gyrus	27	4.441	4	36	-37	56
	L Putamen	5	4.436	4	-24	-7	14
	R Superior Medial Gyrus	5	3.979	3	9	65	26
Turn < Non-Turn	Location not in atlas	13	6.077	5	-6	-13	29
	R Rolandic Operculum	5	5.025	4	42	-16	23
	L Middle Frontal Gyrus	35	4.738	4	-36	53	11
	Location not in atlas	14	4.595	4	30	-25	59
	R Precuneus	15	4.525	4	6	-64	38
	R Middle Frontal Gyrus	14	4.480	4	30	56	5
	R Angular Gyrus	12	4.439	4	36	-67	47
	Location not in atlas	9	4.250	4	-36	-58	35
	Location not in atlas	5	4.125	3	-18	-19	32
	L Middle Frontal Gyrus	5	4.079	3	-36	23	53
	L Inferior Parietal Lobule	5	4.075	3	-51	-55	50
Backtrack > Non-Backtrack (same trial)	R Linual Gyrus	1423	11.736	6	9	-73	5
	Location not in atlas	1045	7.507	5	-27	-13	50
	Location not in atlas	26	6.850	5	-12	-16	-7
	Location not in atlas	61	6.317	5	24	-58	-28
	R Cerebelum (VIII)	47	6.101	5	18	-55	-49
	Location not in atlas	19	5.654	4	-15	-31	38
	R SupraMarginal Gyrus	237	5.600	4	51	-37	38
	Location not in atlas	33	5.244	4	12	-16	2
	R IFG (p. Opercularis)	46	5.241	4	45	14	14
	R Inferior Temporal Gyrus	10	5.064	4	42	-64	-1

	L Thalamus	22	5.039	4	-12	-19	8
	L Middle Frontal Gyrus	28	4.927	4	-39	35	29
	R Middle Frontal Gyrus	17	4.886	4	36	38	26
	Cerebellar Vermis (9)	15	4.885	4	0	-61	-37
	R Cerebelum (Crus 2)	8	4.780	4	45	-52	-34
	Location not in atlas	9	4.389	4	-24	-61	-31
	Location not in atlas	5	4.311	4	15	-31	41
	Location not in atlas	7	4.239	4	-12	20	29
	R Middle Temporal Gyrus	15	4.233	4	42	-70	23
	R Insula Lobe	6	3.972	3	33	17	11
Backtrack > Non-Backtrack (same trial)	R PCC	124	6.643	5	9	-52	29
	L Mid Orbital Gyrus	180	6.353	5	0	62	-10
	L Middle Temporal Gyrus	102	6.189	5	-51	-64	26
	Location not in atlas	34	6.056	5	33	-46	23
	Location not in atlas	29	5.632	4	-12	-16	29
	R Posterior-Medial Frontal	6	5.558	4	12	-28	59
	L Hippocampus	47	5.351	4	-36	-34	-4
	R Amygdala	14	5.131	4	27	-4	-13
	L Middle Orbital Gyrus	8	4.800	4	-27	32	-16
	R Middle Occipital Gyrus	5	4.748	4	30	-94	5
	Location not in atlas	22	4.684	4	-18	-43	20
	Location not in atlas	11	4.664	4	-27	-58	14
	L Superior Frontal Gyrus	7	4.555	4	-21	29	53
	Location not in atlas	26	4.518	4	12	-19	29
	Location not in atlas	7	4.477	4	30	-25	59
	L Middle Temporal Gyrus	27	4.436	4	-60	-40	-1
	R Fusiform Gyrus	5	4.397	4	39	-31	-13
	L Superior Frontal Gyrus	11	4.356	4	-18	41	47
	R Precentral Gyrus	5	4.267	4	39	-25	65
	L Middle Temporal Gyrus	11	4.168	3	-63	-7	-19
	L Superior Medial Gyrus	6	4.062	3	-6	56	35
	Location not in atlas	6	4.020	3	21	-43	17
	R Medial Temporal Pole	8	3.987	3	57	2	-13
	R Superior Frontal Gyrus	7	3.972	3	24	44	44
	R Precentral Gyrus	7	3.968	3	39	-19	47
